# New insights on the Intrinsic, Pro-Apoptotic Effect of IGFB3 in Breast Cancer

**DOI:** 10.3389/fendo.2014.00176

**Published:** 2014-10-21

**Authors:** Antonino Belfiore

**Affiliations:** ^1^Endocrinology, Department of Health Sciences, University Magna Graecia of Catanzaro, Catanzaro, Italy

**Keywords:** IGFBP3, IGF, cancer vaccines, apoptosis, IGF-I receptor

Insulin-like growth factor-binding protein 3 (IGFBP3) is the major carrier of circulating IGF-I and IGF-II. In complexes with ALS, IGFBP3 binds IGFs with high affinity and has a pivotal role in modulating IGFs biological effects by limiting their bioavailability. These ternary complexes are stabilized by IGF-I and ALS, both of which are regulated by the pituitary growth hormone. At the tissue level, however, IGFBP3 is subjected to limited proteolysis by several enzymes, which increase the local IGF bioavailability (1). Besides these “IGF-dependent” IGFBP3 effects, which require a functional IGF-IR, several recent studies have provided convincing evidence that IGFBP3 also elicits significant biological effects in an IGF-independent manner. Indeed, by binding a variety of other molecular partners, located both on cell membrane and in the nucleus, IGFBP3 may modulate cell growth, survival, and transformation (2, 3). Because of these complex activities, IGFBP3 has been considered both a “gatekeeper,” by halting cell proliferation and promoting apoptosis in response to DNA damage, and a “caretaker,” by contributing to DNA repair (2).

Studies focusing on the pro-apoptotic and anti-proliferative actions of IGFBP3 have raised hope that these effects could exploited in cancer therapy, but overall conclusions have remained uncertain (3–5). Even very recent *in vivo* studies have reached discrepant conclusions. By crossing mice with a genetic deletion of IGFBP3 with mice expressing human Myc in the prostate, Metha et al. provided *in vivo* evidence of a role for IGFBP3 as a metastasis suppression gene (6). Accordingly, methylation of the IGFBP3 gene and low IGFBP3 expression occur frequently in aggressive colorectal cancer and are associated with poor response to adjuvant chemotherapy (7). In contrast, in esophageal squamous cell carcinoma, IGFBP3, which is induced by hypoxia, mediates the induction of CD44H cells by suppressing reactive oxygen species (ROS) (8).

Given the context-dependent, multimodal effects of unliganded IGFBP3, mechanistic studies in well-defined cancer cells are therefore needed. In this respect, the study by Perks et al. (9) is a good step in the right direction. In this study, using Hs578T human breast cancer cells, the authors have dissected a signaling cascade activated by IGFBP3, shedding new light on IGF-independent, complex effects of IGFBP3 on apoptosis. As previously described, this cascade is initiated at the membrane level by IGFBP3 binding to caveolin-1 (Cav-1) (10) at the level of Cav-1 scaffolding domain, which also binds and inactivates protein kinase A (PKA) (11). Now, the authors show that, upon binding to Cav-1, IGFBP3 induces Hs578T cell apoptosis by preventing PKA inactivation. Indeed, the PKA inhibitor KT5720 was able to revert IGFBP3-induced apoptosis. To better elucidate this pathway, the Authors investigated a possible role of Rho in this pathway. The effects of Rho are regulated by phosphorylation by a specific serine/threonine kinase (ROCK). Notably, the inhibition of ROCK not only blocked the apoptotic effect of IGFBP3, but enabled IGFBP3 to act as a survival factor, confirming an important role for Rho. Using ROCK inhibitors, it was possible to establish that Rho is downstream of the Cav-1/PKA complex. Previously, the same authors had shown that IGFBP3 binds also to beta 1 integrin (10), which now may be considered, together with Cav-1, upstream to the PKA/ROCK/Rho pathway. To add complexity to this model, the authors also showed that this pathway affects the so-called phospholipid rheostat, which regulates the balance between the pro-apoptotic ceramide and the pro-survival sphingosine-1-phosphate (S1P). In fact, Rho was found to positively regulate ceramide production, which in turn was involved in MAPK activation and apoptosis. Indeed, ceramide production is essential to the apoptotic effect of IGFBP3 along this pathway. However, S1P is enhanced by various growth factors including EGF, IGF-1, and insulin, which activate the S1P kinase, SphK1 (12). IGFBP3 itself may stimulate SphK1 and favor S1P synthesis, which in turn may transactivate EGFR and IGF-IR (13, 14). Therefore, in conditions in which ceramide synthesis is impaired, IGFBP3 may shift the ceramide to S1P balance in favor of S1P and induce survival. This paper of Perks et al. has the merit of having dissected a major pathway activated by unliganded IGFBP3, which may induce both pro-apoptotic and pro-survival effects in a context-specific manner.

Several questions still remain open. Firstly, the context-specific variables that affect the balance between these two opposite effects are still largely unknown. The extracellular matrix composition may certainly be a variable through the involvement of integrins. Indeed, the pro-apoptotic effect of IGFBP3 could be reversed by disrupting integrin receptor complexes (10). The expression level of Cav-1 in cancer cells might also be a variable. It should be mentioned that of Cav-1 is mostly overexpressed during neoplastic progression, and that its role in cancer biology appears to be multidimensional, as it may promote tumorigenesis and metastasis, especially in most advanced cancers, while behaving as an anti-oncogene in other contexts (15). Whether tumor stage may also influence IGFBP3 action is poorly understood.

Secondly, cancer associated fibroblasts (CAFs) may also express dysregulated Cav-1 and IGF system components. Interestingly, elevated IGFBP3 has been shown to play a crucial role in fibroblast-to-myofibroblast differentiation in high-grade prostate cancer (16). Moreover, loss of Cav-1 in CAFs seems to be a marker of oxidative stress and hypoxia and, in breast cancer, is associated with poor clinical outcome (17). One may wonder how Cav-1 and IGFBP3 interact in CAFs.

A third question to be answered is how the tumor microenvironment may influence IGFBP3 expression, and how the effects of IGFBP3 in CAFs and epithelial cells cooperate.

Finally, the interplay between IGF-dependent and IGF-independent effects of IGFBP3 is difficult to address, and is likely to be context-dependent. Although it might be reasonable to expect that a tumor addicted to IGF-IR/IGFs or IR-A/IGF-2 circuits may favorably respond to IGF-blocking activity of IGFBP3, it should be considered that high levels of IGFs and IGFBP3 may switch the balance of the phospholipid rheostat in favor of SP1 by activating SphK1. In this context, the concomitant inhibition of SphK1 should perhaps be taken into consideration.

Overall, it appears that understanding the diverse abilities of IGFBP3 is certainly not straightforward, and more mechanistic studies, possibly on breast cancer stem cells, are needed to establish whether IGFBP3 could be exploited in breast cancer therapy.

## Conflict of Interest Statement

The author declares that the research was conducted in the absence of any commercial or financial relationships that could be construed as a potential conflict of interest.
